# E3 ubiquitin ligase RNF128 negatively regulates the IL-3/STAT5 signaling pathway by facilitating K27-linked polyubiquitination of IL-3Rα

**DOI:** 10.1186/s12964-024-01636-4

**Published:** 2024-05-03

**Authors:** Jingge Yu, Jianguo Li, Ao Shen, Zhiping Liu, Tian-Sheng He

**Affiliations:** 1 School of Basic Medicine, Gannan Medical University, Ganzhou, Jiangxi 341000 China; 2 Center for Immunology, Key Laboratory of Prevention and Treatment of Cardiovascular and Cerebrovascular Diseases, Ministry of Education, Gannan Medical University, Ganzhou, Jiangxi China; 3 School of Graduate, Gannan Medical University, Ganzhou, Jiangxi China; 4 Department of Blood Transfusion, Jingmen Central Hospital, Jingmen, China

**Keywords:** Inflammation, IL-3, RNF128, Ubiquitination, Lysosomal degradation

## Abstract

IL-3/STAT5 signaling pathway is crucial for the development and activation of immune cells, contributing to the cellular response to infections and inflammatory stimuli. Dysregulation of the IL-3/STAT5 signaling have been associated with inflammatory and autoimmune diseases characterized by inflammatory cell infiltration and organ damage. IL-3 receptor α (IL-3Rα) specifically binds to IL-3 and initiates intracellular signaling, resulting in the phosphorylation of STAT5. However, the regulatory mechanisms of IL-3Rα remain unclear. Here, we identified the E3 ubiquitin ligase RNF128 as a negative regulator of IL-3/STAT5 signaling by targeting IL-3Rα for lysosomal degradation. RNF128 was shown to selectively bind to IL-3Rα, without interacting with the common beta chain IL-3Rβ, which shares the subunit with GM-CSF. The deficiency of *Rnf128* had no effect on GM-CSF-induced phosphorylation of Stat5, but it resulted in heightened Il-3-triggered activation of Stat5 and increased transcription of the *Id1*, *Pim1*, and *Cd69* genes. Furthermore, we found that RNF128 promoted the K27-linked polyubiquitination of IL-3Rα in a ligase activity-dependent manner, ultimately facilitating its degradation through the lysosomal pathway. RNF128 inhibited the activation and chemotaxis of macrophages in response to LPS stimulation, thereby attenuating excessive inflammatory responses. Collectively, these results reveal that RNF128 negatively regulates the IL-3/STAT5 signaling pathway by facilitating K27-linked polyubiquitination of IL-3Rα. This study uncovers E3 ubiquitin ligase RNF128 as a novel regulator of the IL-3/STAT5 signaling pathway, providing potential molecular targets for the treatment of inflammatory diseases.

**Supplementary Information:**

The online version contains supplementary material available at 10.1186/s12964-024-01636-4.

## Introduction

Interleukin-3 (IL-3), functioning as a hematopoietic growth factor, not only plays a pivotal role in hematopoiesis regulation but also contributes significantly to immune responses and inflammation. IL-3 influences the growth and differentiation of immune cell populations, particularly impacting the activation of monocytes and granulocytes. IL-3 plays a contributory role in fostering the pro-inflammatory response by boosting the activation and functionality of immune cells. IL-3 has been associated with immune disorders such as sepsis [1], colitis [2, 3], lupus nephritis [4] and arthritis [5]. In sepsis, IL-3 exacerbates acute inflammation by fueling a cytokine storm, and elevated IL-3 levels serve as a predictive indicator of mortality [1]. In inflammatory bowel diseases (IBD), the influence of IL-3 varies based on the degree of intestinal inflammation, which provides protective effects at the initiation of colitis but becomes detrimental during acute colitis [2]. IL-3 also serves as a predictive marker for the severity and outcome of SARS-CoV-2 infection by promoting the recruitment of antiviral plasmacytoid dendritic cells [6]. The IL-3 signaling pathway transmitted by IL-3 receptor complex, which comprises the IL-3 receptor alpha-chain (IL-3Rα, CD123) possessing specificity for IL-3 binding and the common beta chain (IL-3Rβ, CD131) for granulocyte-macrophage colony-stimulating factor (GM-CSF) and IL-5 [7, 8]. Upon IL-3 binding to its receptor, Janus kinases, particularly JAK2, are recruited to the receptor complex and phosphorylate specific tyrosine residues on transcription factor signal transducer and activator of transcription 5 (STAT5). Phosphorylated STAT5 form dimers and translocate to the cell nucleus, regulating cell survival and activation [9].

RNF128, also identified as GRAIL (gene related to energy in lymphocytes), belongs to the family of RING-domain-containing E3 ligases and has been demonstrated to play a role in both innate and adaptive immunity. RNF128 was initially discovered to regulate the induction of naive T cell tolerance and the function of Treg cells by promoting the ubiquitination of CD3 and the degradation of TCR-CD3 [10]. RNF128 was found associated with the disruption of IL2 transcription and the inhibition of CD4^+^ T cell proliferation, leading to the induction of an anergy phenotype through disruption of T cell stimulatory signaling [11]. RNF128 also promotes the ubiquitination and degradation of IL-21R, consequently dampening the cytotoxicity of CD8^+^ T cells and expediting the development of neoplastic lesions in lymphoma [12]. Subsequently, RNF128 was found to exert opposing functions in innate immunity. RNF128 acts as a positive regulator in the immune response induced by RNA/DNA viruses by directly interacting with TBK1 and catalyzing the K63-linked polyubiquitination of TBK1 [13]. RNF128 emerges as a pivotal regulator in acute infectious diseases, as evidenced by its heightened expression during the onset of the acute phase of lung injury [14, 15]. Notably, RNF128 knockout mice were observed higher pulmonary edema and inflammatory cell infiltration when compared to their wild-type counterparts following induction of acute lung injury [15]. Upon recovery of ulcerative colitis (UC) patients with elevated clinical activity index scores, the heightened expression of RNF128 suggests that it contributes to improved disease remission [16]. Therefore, RNF128 exhibits a characteristic of alleviating excessive inflammatory responses and organ damage in inflammatory diseases, independent of its role in positively regulating the immune response to type I interferons. The function and mechanism of RNF128 in the early stages of inflammatory responses remain unclear.

In this study, we reported that RNF128 as a negative modulator of IL-3/STAT5 signaling to suppress inflammatory responses. RNF128 was significantly upregulated in response to IL-3 stimulation and exhibited specific binding to IL-3 receptor alpha chain IL-3Rα, rather than the common beta chain IL-3Rβ. The deficiency of *Rnf128* did not impact the GM-CSF-induced phosphorylation of Stat5 but led to enhanced Il-3-triggered activation of Stat5 and increased transcription of the *Id1*, *Pim1*, and *Cd69* genes. RNF128 facilitates the K27-linked polyubiquitination of IL-3Rα, ultimately promoting its degradation through the lysosomal pathway. Conversely, RNF128 inhibits the activation and chemotaxis of macrophages in response to LPS stimulation, thereby attenuating excessive inflammatory responses. In summary, our findings revealed a novel role of RNF128 in controlling IL-3-triggered inflammatory responses through the mediation of K27-linked polyubiquitination and subsequent degradation of IL-3Rα.

## Materials and methods

### Mice

Wild-type (WT) and *Rnf128*-deficient mice on a C57BL/6 background were generously provided by Dr. Chengjiang Gao (Shandong University, Jinan, China) [13]. Genotyping of WT and *Rnf128*^*−/−*^ mice was performed with primers: forward primer, 5′-GCTGAAGTTAGTACTGCATG-3′ and reverse primer 5′-GTCAACAGGTGGCAGATACC-3′. The mice were housed in specific pathogen-free (SPF) conditions at the Experimental Animal Center of Gannan Medical University, with access to sterile water and food. Mice aged over 8 weeks, matched for age and sex, were utilized for all experiments. The Institutional Research Ethics Committee of Gannan Medical University approved this study.

### Cell culture and constructs

HEK293 and HeLa cells were obtained from the American Type Culture Collection and cultured in DMEM (Biological Industries) supplemented with 10% FBS (Biological Industries). Human TF-1 cells, generously provided by Procell Life Science and Technology, were cultured in RPMI-1640 (Corning) medium supplemented with 2 ng/mL GM-CSF and 10% FBS. To obtain and culture bone marrow-derived macrophages (BMDMs), mice were sacrificed, and femurs and tibia were aseptically removed. Bone marrow cells were then flushed from the femur and tibia and cultured in 150-mm dishes with 15 mL of IMDM medium supplemented with 10% FBS and 20 ng/mL M-CSF for a duration of 6 days. All cells were incubated in a cell culture chamber at 37 °C with 5% CO_2_. Transient transfection for HEK293 and HeLa cells were performed using polyethylenimine (PEI; Polysciences, Warrington, PA, USA) as previously described [17].

Plasmids expressing Flag- or HA-tagged RNF128 for mammalian expression were generated using standard molecular cloning methods. The pcDNA3.1-IL-3Rα-Flag and pcDNA3.1-IL-3Rβ-Flag were obtained from Changsha You Bao Biotechnology Co. Ltd. (Changsha, China). Ubiquitin and its mutant ubiquitin plasmids (K6, K11, K27, K29, K33, K48 and K63) were a gift from Dr. Liang-Guo Xu (Jiangxi Normal University, Nanchang) [18, 19]. The mutant ubiquitin plasmids (K6R, K11R, K27R, K29R, K33R, K48R and K63R) were purchased from MiaoLingPlasmid (Wuhan, China). Human RNF128-shRNA constructs were generated by cloning the following double-stranded oligonucleotides into the pLKO.1 vector. The designed shRNA sequences targeting RNF128 included: scramble shRNA, 5′-AATGCACGCTCAGCACAAGC-3′; RNF128 shRNA, 5′-AGAGACTGCTGTTCGAGAAAT-3′.

### Reagents and antibodies

Primary antibodies used in this study included Anti-IL-3Rα antibody (ABclonal, A3926), anti-STAT5 antibody (Cell Signaling Technology, 94205), anti-phospho-STAT5 antibody (Cell Signaling Technology, 4322), anti-β-actin antibody (Proteintech Group, 66009-1-Ig), anti-RNF128 antibody (Cell Signaling Technology, 71,590), anti-LC3B antibody (Cell Signaling Technology, 3868), anti-Flag-tag antibody (Sigma-Aldrich, F1804), anti-HA-tag antibody (Invitrogen, 26,183), and anti-Myc-tag antibody (Proteintech Group, 16286-1-AP) were employed. Secondary antibodies included HRP conjugated goat anti-rabbit IgG (Boster Bio, BA1054), HRP conjugated goat anti-mouse IgG (Boster Bio, BA1050), TRITC conjugated goat anti-mouse IgG (Boster Bio, BA1089), DyLight®488 conjugated goat anti-mouse IgG (Boster Bio, BA1126), TRITC conjugated goat anti-rabbit IgG (Boster Bio, BA1090), and DyLight®488 conjugated goat anti-rabbit IgG–488 (Boster Bio, BA1127). All antibodies were purchased from the respective suppliers as indicated. Recombinant human IL-3 (Pepro Tech, 200-03), mouse Il-3 (MCE, HY-P7062), mouse GM-CSF (Pepro Tech, 213–13) and LPS (Sigma-Aldrich, L2880) were purchased from the respective suppliers. MG132 (HY-13259), Chloroquine (HY-17589 A), and Cycloheximide (HY-12320) were obtained from MCE (MedChemExpress).

### RT-qPCR

Total RNA extraction was carried out using TransZol-Up (TransGen Biotech, ET111-v2) and resuspended in RNase-free water following the manufacturer’s protocols. For cDNA synthesis, 1 µg of total RNA was reverse transcribed into complementary DNA using PrimeScript™ RT Master Mix (RR036A, Takara). Gene expression levels were analyzed by real-time qPCR using a SybrGreen I-based kit (TransGen Biotech, AQ601-02-V2) with gene-specific primers. The housekeeping gene GAPDH served as the internal reference to normalize for variations in RNA input and cDNA synthesis efficiency. The threshold cycle (Ct) values were determined for each sample and the ΔΔCt method was utilized for the quantitative analysis of relative gene expression. Primer sequences used in mice are as follows: Pim1-F: 5’-TGTCTCTTCAGAGTGTCAGC-3’, Pim1-R:5’-CGGATTTCTTCAAAGGAGGG-3’; Cd69-R: 5’-GTAGCAACATGGTGGTCAG-3’; Id1-F: 5’-AACTCGGAGTCTGAAGTCG-3’, Id1-R: 5’-GACACAAGATGCGATCGTC-3’; Il6-F: 5’-TAGTCCTTCCTACCCCAATTTCC-3’, Tnfα-F: 5’-CCCTCACACTCAGATCATCTTCT-3’, Tnfα-R: 5’-GCTACGACGTGGGCTACAG-3’; Cd69-F: 5’-TCTCATTGCCTTAAATGTGGG-3’, Il6-R: 5’-TTGGTCCTTAGCCACTCCTTC-3’; Kc-F: 5’-CTGGGATTCACCTCAAGAACATC-3’, Kc-R: 5’-CAGGGTCAAGGCAAGCCTC-3’; Ccl2-F: 5’-TTAAAAACCTGGATCGGAACCAA-3’, Ccl2-R: 5’-GCATTAGCTTCAGATTTACGGGT-3’; Ccl5-F: 5’-GCTGCTTTGCCTACCTCTCC-3’, Ccl5-R: 5’-TCGAGTGACAAACACGACTGC-3’; Cxcl10-F: 5’-CCAAGTGCTGCCGTCATTTTC-3’, Cxcl10-R: 5’-GGCTCGCAGGGATGATTTCAA-3’; Gapdh-F: 5’-AGGTCGGTGTGAACGGATTTG-3’, Gapdh-R: 5’-TGTAGACCATGTAGTTGAGGTCA-3’. Primer sequences used in human cells are as follows: CD69-F: 5’-GCTGGACTTCAGCCCAAAATGC-3’, CD69-R: 5’-AGTCCAACCCAGTGTTCCTCTC-3’; PIM1-F: 5’-TCTACTCAGGCATCCGCGTCTC-3’, PIM1-R: 5’-CTTCAGCAGGACCACTTCCATG-3’; ID1-F: 5’-GTTGGAGCTGAACTCGGAATCC-3’, ID1-R: 5’-ACACAAGATGCGATCGTCCGCA-3’; GAPDH-F: 5’-AGCCTCAAGATCATCAGCAATG-3’, GAPDH-R: 5’-ATGGACTGTGGTCATGAGTCCTT-3’.

### Immunofluorescence staining

Immunofluorescence staining was performed as previously described [17]. Briefly, HeLa cells were fixed with 4% paraformaldehyde for 15 min, permeabilized with 0.1% Triton X-100, and subsequently blocked by 3% BSA for 2 h to prevent nonspecific binding. Incubation with primary antibodies against the target proteins was carried out overnight at 4 °C, followed by appropriate fluorophore-conjugated secondary antibodies before fluorescence microscopy. Nuclei were visualized by counterstaining with DAPI, and imaging was conducted using a laser scanning confocal fluorescence microscope (ZeissLSM880), equipped with filters suitable for the respective fluorophores.

### Co-IP and immunoblotting

Co-IP and Immunoblotting experiments were performed as previously described [18, 20]. Briefly, 60 µL of protein A agarose resin (Yeasen Biotechnology) was incubated for 12 h with 0.4 µg of anti-Flag antibody for each immunoprecipitation and cell lysate by RIPA at 4 °C. After incubation, the agarose resin was subjected to wash with ice-cold lysis buffer. The immunoprecipitated samples were then analyzed using immunoblotting with the indicated antibodies. For immunoblotting, cells were lysed using RIPA buffer supplemented with protease and phosphatase inhibitors (PMSF, cocktail). Subsequently, protein samples were separated via 12% SDS-PAGE and electrophoretic transferred onto PVDF membranes (Millipore). These membranes were then blocked with 5% nonfat milk for 1 h, followed by an overnight incubation with primary antibodies at 4 °C. After thorough washing, the membranes underwent a 1-hour incubation with secondary antibodies. Protein visualization was achieved using an ECL chemical fluorescence chromogenic solution from Thermo Scientific, and the results were analyzed using ImageJ software.

### Transwell migration assay

Transwell chambers (Corning) were used for Transwell migration assay to measure the migratory response of BMDMs. Single-cell suspension of BMDMs were seeded in the upper chamber of transwell (pore size: 8 μm) at a density of 1 × 10^5^ cells in 200 µL culture medium. The lower chamber contained culture medium with or without the addition of LPS or LPS/Il-3. After 36 h of incubation, cells were fixed in 4% paraformaldehyde, stained with 0.2% crystal violet. Non-migratory cells on the upper chamber were gently removed with a cotton swab. Transwell inserts were observed and photographed under a microscope, and the results were quantified using Image J software.

#### LC-MS/MS

Human TF-1 cells (~ 4 × 10^6^) were infected with either pCDH-HA-RNF128 or pCDH lentivirus by gently centrifugation at 800 rpm for 90 min. The culture media was replaced with fresh growth medium 24 h after infection and the cells were harvested at 48 h post-infection. Cell lysis was achieved by RIPA buffer for 30 min on ice and centrifuged at 12,000 × g for 8 min at 4 °C. The supernatant was incubated overnight with anti-HA agarose beads at 4 °C. The next day, beads were washed by cold 1 M NaCl RIPA buffer for 10 min and three times with PBS to remove nonspecific proteins. Proteins were solubilized by resuspending in SDS loading buffer and boiled for denaturation. Subsequently, the eluted proteins in the supernatant were analyzed by LC-MS/MS.

### Statistical analysis

Statistical analysis was performed using GraphPad prism 9.0 (GraphPad Prism, San Diego, CA, USA), and the presented data are expressed as the mean ± standard error of the mean (SEM). To assess the statistical significance of variances between two groups, an unpaired Student’s t-test was employed, while differences among multiple groups were evaluated through ANOVA analysis. A significance level of *P < 0.05 was employed to denote statistical significance, indicating a noteworthy difference in the observed results.

## Results

### RNF128 interacts with IL-3 receptor IL-3Rα

**Fig. 1:**
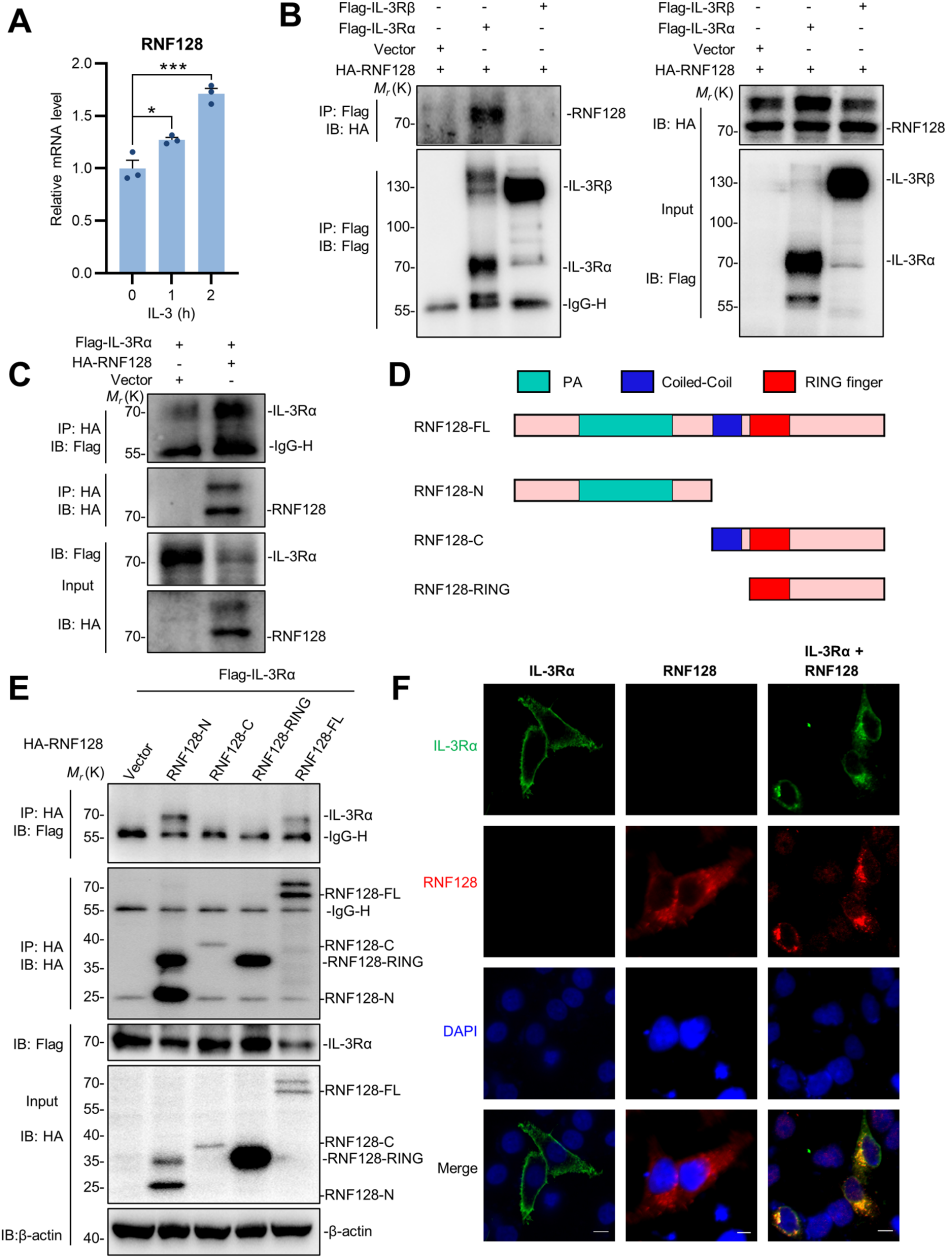
RNF128 interacts with IL-3 receptor IL-3Rα. (**A**) RNF128 expression levels significantly elevated upon IL-3 treatment. THP-1 cells were treated with IL-3 for 1 or 2 h, and the expression of RNF128 was evaluated using qPCR. (**B**) RNF128 interacts with IL-3Rα, not IL-3Rβ. 293T cells were transfected with HA-RNF128, along with Flag-tagged IL-3Rα and IL-3Rβ, and lysed after 24 h. The lysates subjected to immunoprecipitation using an anti-Flag antibody, followed by immunoblotting with the indicated antibodies. (**C**) The reverse interaction was confirmed by immunoprecipitation using an anti-Myc antibody in cells co-expressing Myc-RNF128 and Flag-IL-3Rα. (**D**, **E**) The N-terminal domain of RNF128 is primarily responsible for binding to IL-3Rα. 293T cells were transfected with Flag-IL-3Rα and HA-tagged truncated form of RNF128, and the immunoprecipitation analysis was conducted using an anti-Flag antibody. (**F**) Analysis of the colocalization between RNF128 and IL-3Rα. HeLa cells co-expressing RNF128 and IL-3Rα were subjected to immunostaining, with RNF128 detected using FITC and IL-3Rα using TRITC. Fluorescence signals were observed using confocal laser scanning microscopy. Scale bars: 10 μm. The data are presented as the mean ± SEM. * *P* < 0.05; ** *P* < 0.01; *** *P* < 0.001

RNF128 has been demonstrated to function oppositely in both innate and adaptive immunity. Through mass spectrometry analysis of proteins bound to RNF128, we identified the IL-3 receptor (P269511, IL-3 receptor subunit alpha) in our screening process (Table S1). IL-3 is an essential cytokine that regulates various aspects of hematopoiesis and immune cell functions. To investigate the effects of RNF128 on IL-3 signaling, we initially analyzed the level of RNF128 following IL-3 stimulation. The results indicated that the mRNA of RNF128 significantly increased upon IL-3 treatment (Fig. 1A), suggesting a close association between RNF128 and IL-3 signaling. Furthermore, Co-IP assays were conducted by overexpressing exogenous HA-RNF128 and Flag-tagged IL-3 receptors. The results revealed that IL-3 receptor alpha chain IL-3Rα, rather than the common beta chain IL-3Rβ, specifically interacts with RNF128 (Fig. 1B), implying a specific function of RNF128 in IL-3-mediated cellular signaling. The association between IL-3Rα and RNF128 was further confirmed through reverse co-immunoprecipitation experiments (Fig. 1C). Considering that the E3 ubiquitin-protein ligase RNF128 primarily comprises three structural domains, including the N-terminal protease-associated domain (PA), coiled-coil region, and a zinc RING finger domain. To identify the specific region responsible for the interaction, we generated a series of expression fragments of RNF128 based on its domain boundaries (Fig. 1D). As shown in the Fig. 1E, the N-terminal domain of RNF128 is primarily responsible for binding to IL-3Rα. Furthermore, we also analyzed the subcellular colocalization of RNF128 and IL-3Rα using confocal microscopy. Under physiological conditions, IL-3Rα is predominantly expressed on the cell membrane. However, when co-expressed with RNF128, IL-3Rα predominantly translocates into the cytoplasm and exhibits pronounced colocalization in distinct puncta with RNF128 (Fig. 1F). Therefore, the E3 ubiquitin ligase RNF128 is likely to regulate IL-3-mediated signaling by interacting with IL-3Rα.

### RNF128 mediates the degradation of IL-3Rα via lysosomal pathway

**Fig. 2:**
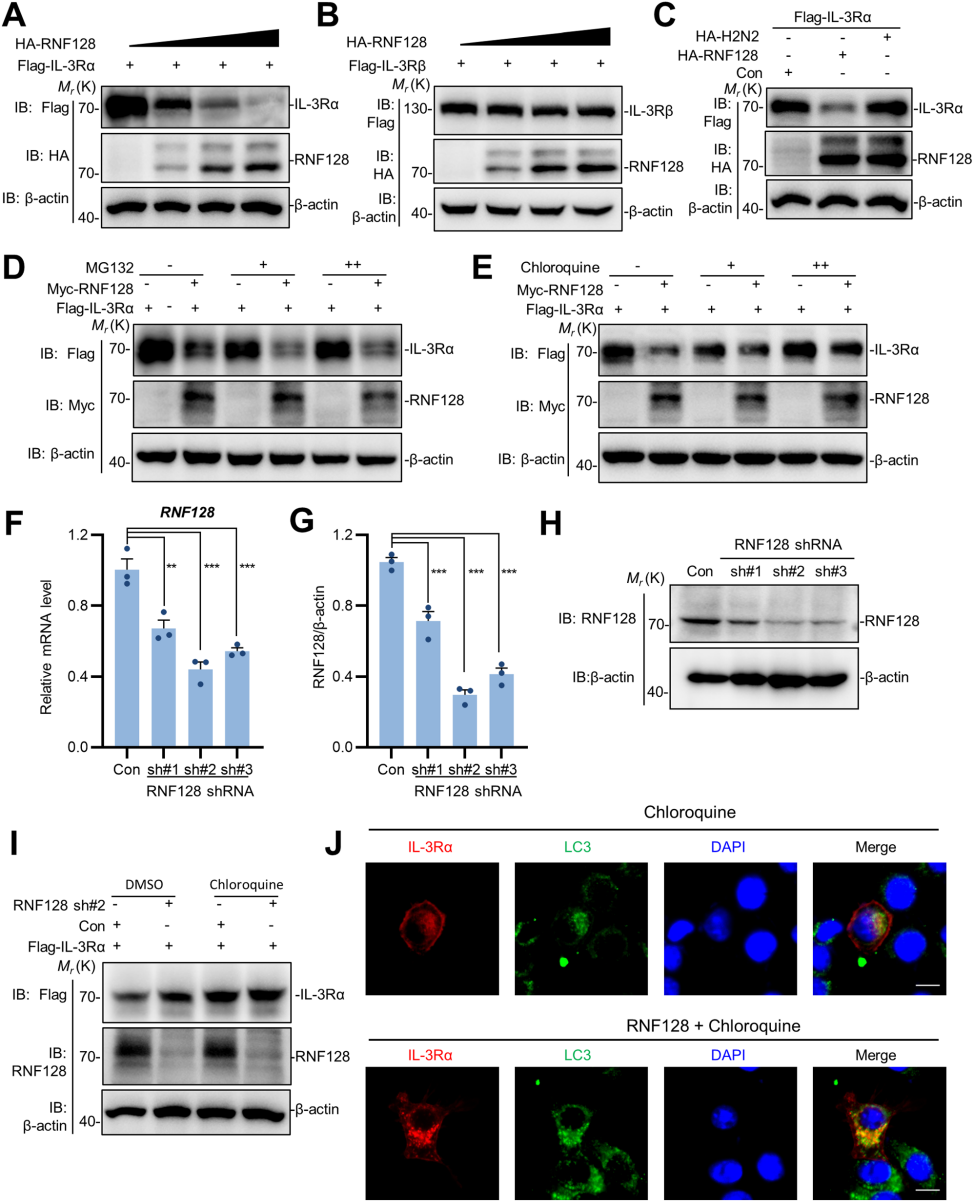
RNF128 mediates the degradation of IL-3Rα via lysosomal pathway. (**A**-**B**) The effect of RNF128 overexpression on IL-3Rα and IL-3Rβ. 293T cells were plated in 6-well plates and co-transfected with Flag-tagged IL-3Rα (**A**) or IL-3Rβ (**B**) along with varying quantities of HA-RNF128 (0, 0.2, 0.4 and 0.8 µg). (**C**) E3 ubiquitin ligase activity of RNF128 influences the stability of IL-3Rα. 293T cells were transfected with Flag-IL-3Rα, along with either wild-type RNF128 or a ligase-inactive mutant (HA-H2N2), and the expression of IL3Rα was analyzed by immunoblotting. (**D**, **E**) RNF128 mediated lysosome-dependent degradation of IL-3Rα. 293T cells were transfected Myc-RNF128 with and Flag-IL-3Rα for 10 h, followed by treatment with CHX, MG132, or chloroquine for 6 h before assessing the levels of IL3RA by immunoblotting. (**F**) Knockdown of RNF128 expression by specific shRNAs using qPCR. (**G**, **H**) The inhibitory effects of specific shRNAs on RNF128 expression were assessed by immunoblotting. (**I**) The effects of RNF128 knockdown on the stability of IL3Rα. HCT116 cells were transfected with Flag-IL-3Rα and RNF128 shRNA#2, followed by treatment with DMSO or chloroquine for 6 h, and the expression levels of IL3Rα were assessed. the colocalization of LC3 and IL-3Rα in the presence or absence of RNF128. HeLa cells were co-transfected with IL-3Rα, and immunostaining was performed to assess the colocalization of LC3 and IL-3Rα in the presence or absence of RNF128. IL-3Rα using TRITC and LC3 detected using FITC. Scale bars: 10 μm. The data are presented as the mean ± SEM. * *P* < 0.05; ** *P* < 0.01; *** *P* < 0.001

Upon IL-3 stimulation, the IL-3 receptor IL-3Rα becomes activated and performs its function by recruiting interacting proteins; concurrently, it undergoes degradation in a time-dependent manner following IL-3 treatment (Figure S1A-B), which subsequently modulates the downstream signaling pathways. Interestingly, we observed that overexpression of full-length RNF128 attenuates the protein expression of IL-3Rα in the input samples (Fig. 1C and E). Considering that RNF128 is an E3 ubiquitin ligase with a RING domain, we further explored its potential effect on the protein stability of IL-3 receptor by incrementally increasing the dosage of RNF128. The results overexpression of RNF128 significantly facilitated degradation of IL-3Rα in a dose-dependent manner (Fig. 2A), whereas RNF128 did not exhibit a noticeable degradation effect on IL-3Rβ (Fig. 2B). We generated an enzymatically inactive mutant of the RNF128 ubiquitin ligase, termed RNF128-H2N2, by substituting the conserved histidine residue with an asparagine in the RING finger domain [21, 22]. The findings revealed that the levels of IL-3Rα were reduced by the wild-type RNF128 but not by the ligase-inactive mutant (Fig. 2C), suggesting that its E3 ubiquitin ligase activity is essential for the stability of IL-3Rα. Studies indicate that proteins are primarily degraded through two pathways: the proteasomal degradation pathway and the lysosomal degradation pathway [23, 24]. We observed that the degradation of IL-3Rα mediated by RNF128 was not affected by the proteasomal inhibitor MG132 (Fig. 2D); however, it could be rescued by treating cells with the lysosomal inhibitor chloroquine (CQ) (Fig. 2E). The results suggested that RNF128 influenced the stability of IL-3Rα through lysosome-dependent degradation pathway.

To investigate the potential roles of endogenous RNF128 in influencing the stability of IL-3Rα, we subsequently screened for specific and effective RNF128 shRNA to suppress its native expression. We designed three RNF128 shRNAs for targeting *RNF128* mRNA and evaluated their efficiency using Western blotting and real-time qPCR. We observed that the three RNF128 shRNAs construct exerted varying effects on the expression of RNF128. Among them, RNF128shRNA#2 exhibited the most effective knockdown efficiency for reducing RNF128 expression, so we chose this for subsequent experiments (Fig. 2F-H). Consistent with the overexpression results, knockdown of RNF128 resulted in an increase in IL-3Rα expression, and this effect could be reversed by the lysosomal inhibitor chloroquine (Fig. 2I). LC3 (Microtubule-associated protein 1 A/1B-light chain 3) plays a pivotal role in the autophagic degradation pathway, regulating cellular autophagic activity by participating in the formation and degradation of autophagosomes. Therefore, we further observed the subcellular localization of IL-3Rα and LC3 using immunofluorescence techniques to corroborate our findings. The results indicated that IL-3Rα predominantly localized to the membrane and did not show significant colocalization with LC3. However, upon co-expression with RNF128, IL-3Rα translocated into the cytoplasm, exhibiting noticeable colocalization with LC3 (Fig. 2J). Collectively, RNF128 facilitates the degradation of IL-3Rα through the lysosomal pathway.

### RNF128 negatively regulates IL-3-triggered signaling

**Fig. 3:**
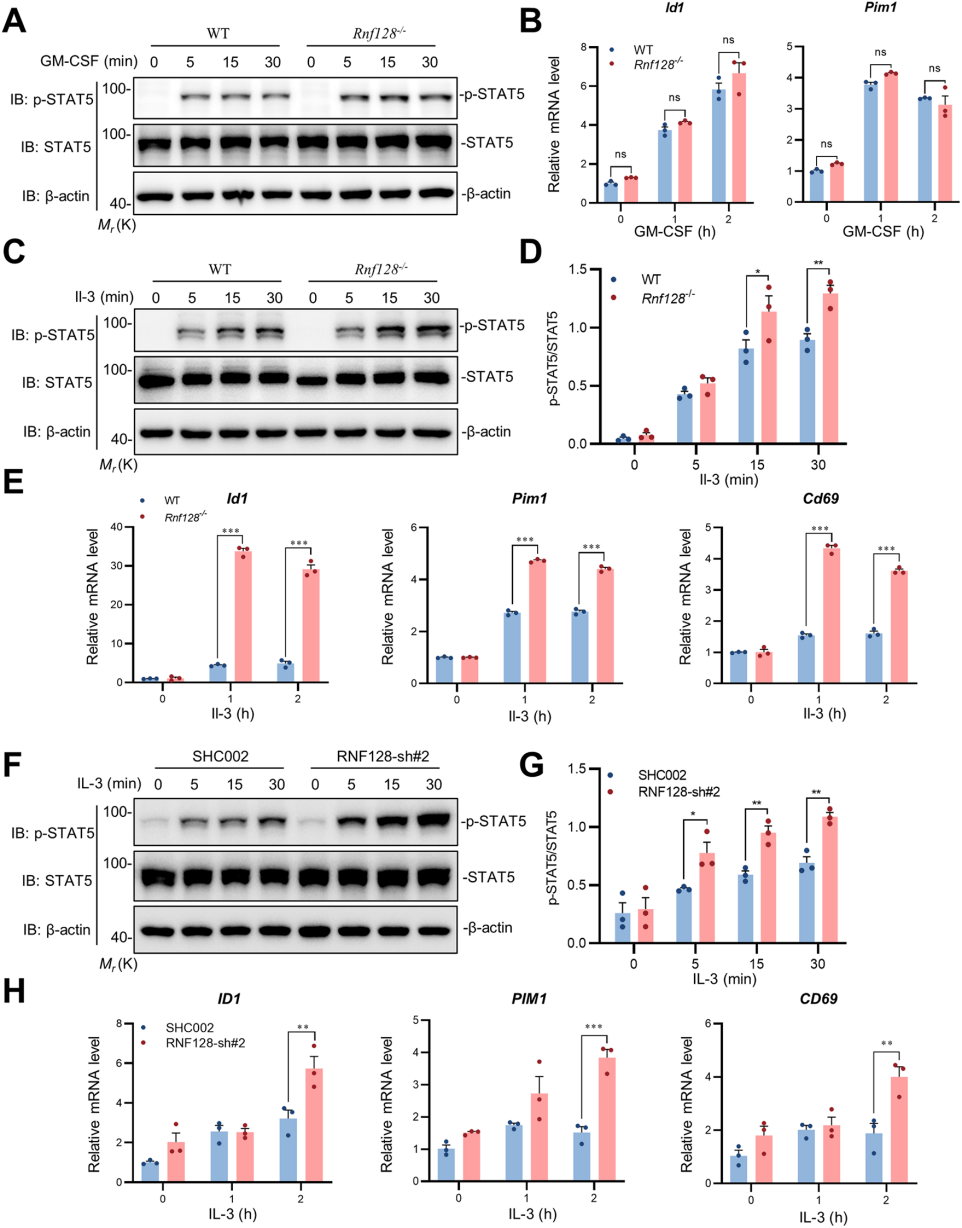
RNF128 negatively regulates IL-3-triggered signaling. (**A**, **B**) Effects of Rnf128 on GM-CSF-induced phosphorylation of Stat5 and transcription of downstream genes. Wild-type and *Rnf128*-deficient BMDMs were treated with GM-CSF (10 ng/ml) or not for the indicated times before immunoblotting analysis and qPCR analysis. (**C**, **D**) Effects of Rnf128-deficient on Il-3-induced phosphorylation of Stat5. Wild-type and *Rnf128*-deficient BMDMs were either untreated or treated with Il-3 (25 ng/mL) for the specified durations. Subsequently, immunoblotting analysis was conducted to detect p-Stat5 levels, with quantification illustrating the ratio of p-Stat5 to Stat5 (**D**). (**E**) Effects of Rnf128-deficient on Il-3-induced *Id1*, *Pim1* and *Cd69* genes. (**F**, **G**) Effects of RNF128 knockdown on IL-3-induced phosphorylation of Stat5. RNF128 knockdown and control TF-1 cells were subjected to overnight starvation, followed by stimulation with IL-3 (20 ng/mL) for the indicated durations. Subsequently, immunoblotting analysis was conducted for p-STAT5, and the quantification of p-STAT5/STAT5 ratios is presented (**G**). (**H**) Effects of RNF128 knockdown on the transcription of the ID1, PIM1 AND CD69 genes induced by IL-3. * *P* < 0.05; ** *P* < 0.01; *** *P* < 0.001. Statistical analysis was performed using two-way ANOVA with Dunnett’s post-hoc test

The IL-3 receptor complex consists of several subunits crucial for triggering cellular responses to IL-3, including IL-3Rα and IL-3Rβ. IL-3Rα is the dedicated subunit that exhibits a high-affinity binding to IL-3, whereas IL-3Rβ is shared with other cytokines, such as GM-CSF and IL-5. Upon engaging with their respective cytokines on the cell surface, the receptors stimulate Janus kinases (JAKs), subsequently leading to the phosphorylation and activation of downstream transcription factor STAT5. To investigate the roles of RNF128 in IL-3-triggered signaling, we initially employed *Rnf128*-deficient mice created through the CRISPR/Cas9 system. The results indicated that the deficiency of Rnf128 did not affect the GM-CSF-induced phosphorylation of Stat5 or the GM-CSF-induced transcription of the *Id1* and *Pim1* genes in BMDMs (Fig. 3A-B). On the contrary, when BMDMs were stimulated with IL-3, the absence of RNF128 markedly enhanced the Il-3-triggered phosphorylation of Stat5 (Fig. 3C-D). Consistently, qPCR analysis revealed that the absence of Rnf128 promoted the transcription of the *Id1*, *Pim1* and *Cd69* genes induced by Il-3(Fig. 3E). This result also confirmed the above data that RNF128 did not bind to IL-3Rβ but interacted exclusively with IL-3Rα. We subsequently investigated the effects of RNF128 on IL-3-triggered signaling in human TF-1 cells. Immunoblotting analysis revealed that knockdown of RNF128 significantly enhanced the activation of STAT5 induced by IL-3 (Fig. 3F). Similarly, knockdown of RNF128 also promoted the IL-3-triggered transcription of the *ID1*, *PIM1* AND *CD69* genes comparing to control TF-1 cells (Fig. 3G). These results suggest that RNF128 does not affect GM-CSF-induced signaling but specifically negatively regulates IL-3-triggered signaling.

### RNF128 facilitates the K27-linked polyubiquitination of IL-3Rα

**Fig. 4:**
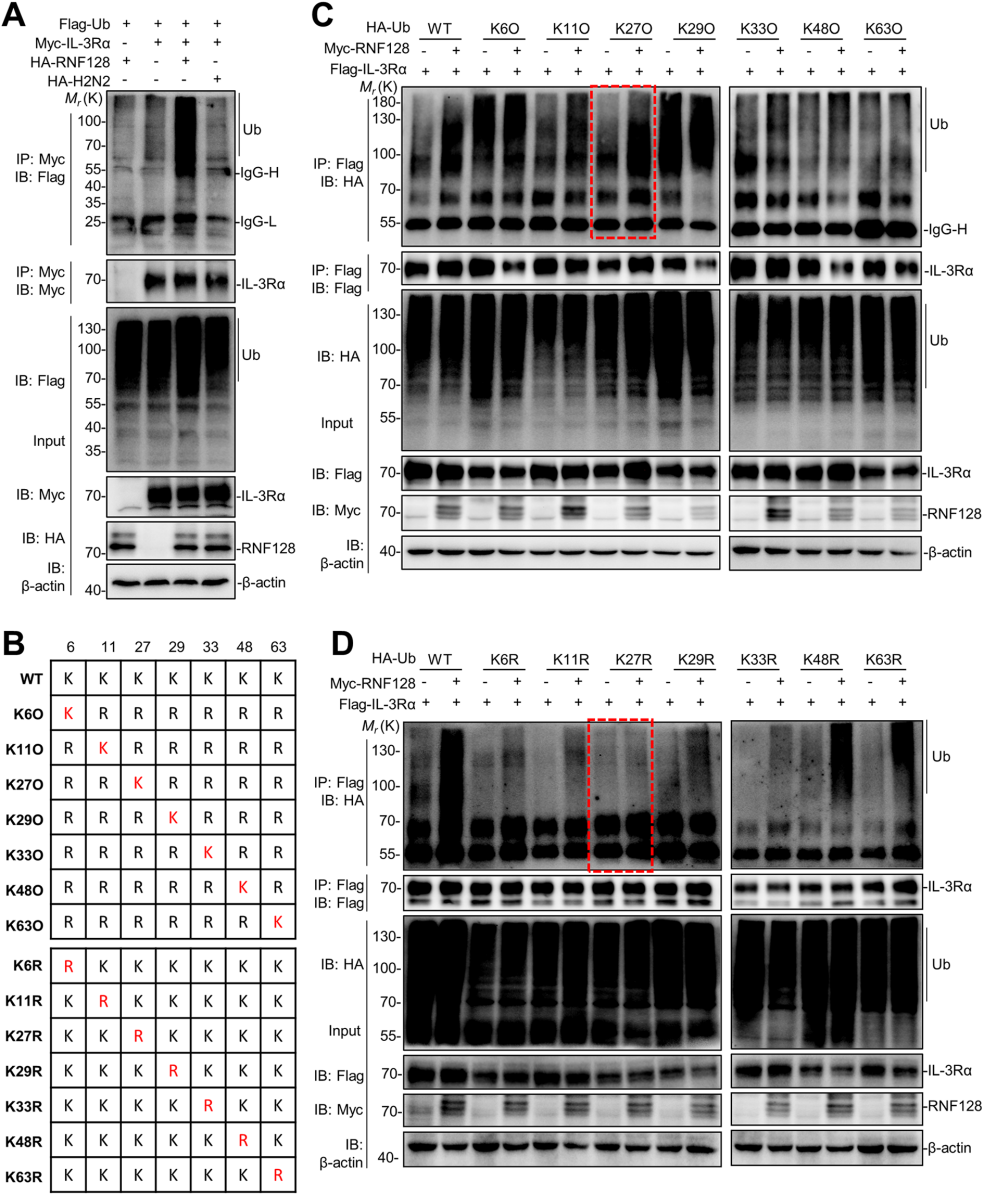
RNF128 facilitates the K27-linked polyubiquitination of IL-3Rα. (**A**) RNF128 significantly enhanced the polyubiquitination of IL-3Rα depending on its ubiquitin ligase activity. 293T cells were transfected with Flag-IL-3Rα, HA-ubiquitin, and either Myc-RNF128 or its ligase-inactive mutant. The cells were treated with chloroquine for 10 h before lysis. Lysates were immunoprecipitated with an anti-Flag antibody and analyzed by immunoblotting with the indicated antibodies. (**B**) Schematic diagram of the various HA- ubiquitin mutants generated by site-directed mutagenesis to replace each lysine in ubiquitin with arginine. (**C**, **D**) RNF128 facilitates the K27-linked polyubiquitination of IL-3Rα. 293T cells underwent transfection with Flag-IL-3Rα, Myc-RNF128, and HA-ubiquitin or its respective mutants (HA-ubiquitin was either preserved with only one remaining lysine residue or subjected to a single lysine mutation). The cells were treated with chloroquine for 10 h before lysed by RIPA lysis buffer. Cell lysates were immunoprecipitated with an anti-Flag antibody and subsequently analyzed by immunoblotting with the indicated antibodies

Ubiquitination serves as a signal for the recognition and targeting of specific substrates for degradation by the autophagy-lysosome pathway. Upon IL-3 stimulation, the level of ubiquitination for IL-3Rα increases significantly in a short time frame (Figure S2). Given that E3 ubiquitin ligase RNF128 promotes the lysosomal degradation of IL-3Rα in a ligase activity-dependent manner, we hypothesized that RNF128 could influence the ubiquitination of the IL-3Rα, thereby facilitating its degradation. Consistently, we found that the wild-type RNF128 significantly increased the polyubiquitination of IL-3Rα, while the ligase-inactive mutant of RNF128 did not affect the polyubiquitination of IL-3Rα (Fig. 4A). Based on the ubiquitin linkage types on the substrates, various forms of ubiquitination have been identified, including K6-, K11-, K27-, K29-, K33-, K48-, and K63-linked polyubiquitination. To further identify which type of ubiquitylation type is essential for RNF128-mediated ubiquitination of IL-3Rα, we employed site-directed mutagenesis to replace each of the seven lysine in ubiquitin with arginine. In these mutants, we either preserved only one lysine residue for ubiquitin linkage or replaced the specified lysine with an arginine (Fig. 4B). As indicated by the results, among all types of polyubiquitin, the K27-linked polyubiquitylation of IL-3Rα was most noticeable increased by the co-expression of RNF128 (Fig. 4C). Conversely, no differences were observed with the ubiquitin(K27R) mutant between the control and co-expression with RNF128, while significant distinctions were maintained for the other ubiquitin mutants (Fig. 4C). Therefore, RNF128 promotes the K27-linked polyubiquitination of IL-3Rα, subsequently facilitating its lysosomal degradation.

### RNF128 knockout promotes the inflammatory process induced by LPS

**Fig. 5:**
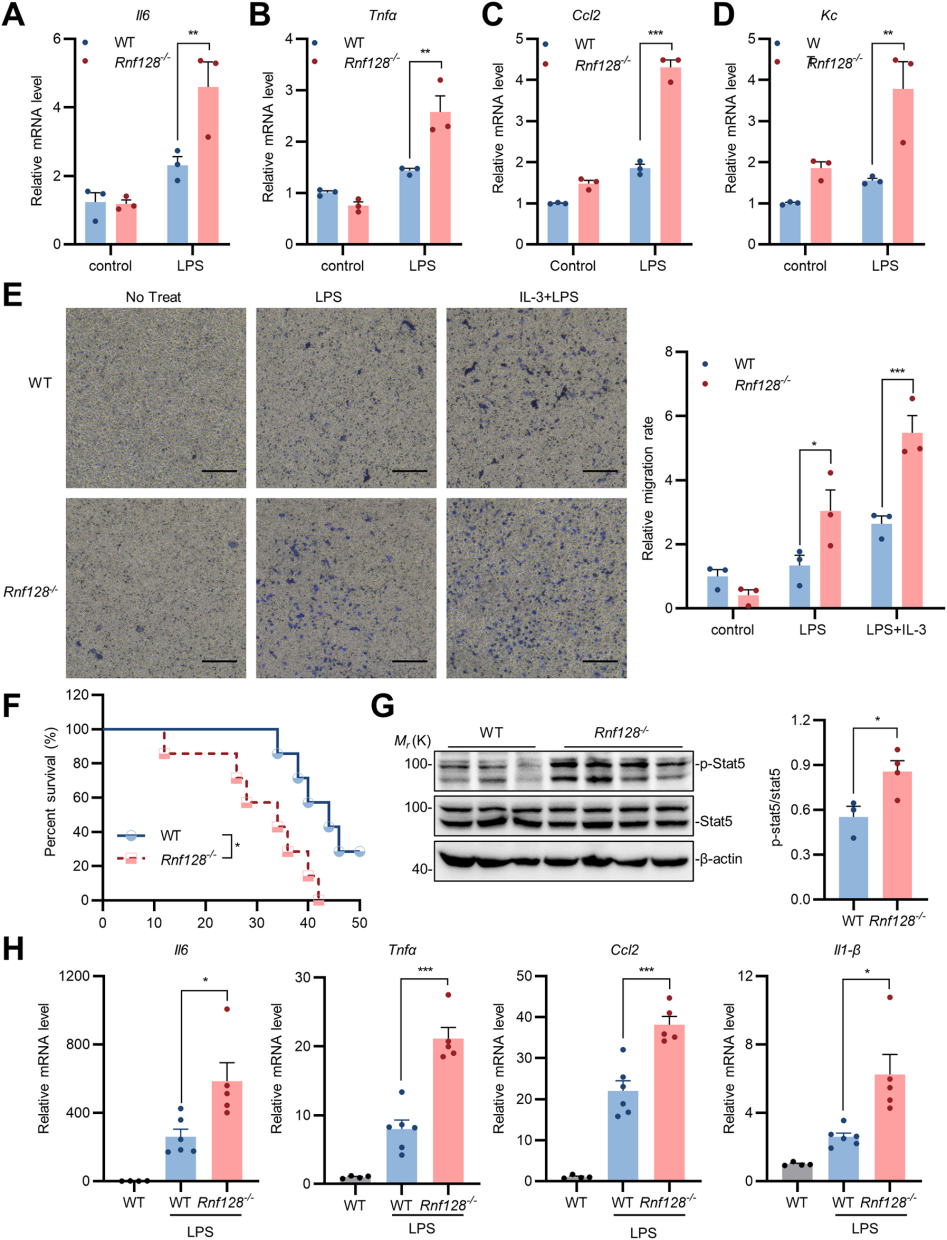
RNF128 knockout promotes the activation and chemotaxis of macrophages to LPS. (**A**-**D**) *Rnf128* deficiency enhanced LPS-induced proinflammatory cytokines. Wild-type and *Rnf128*-deficient BMDMs were either untreated or treated with LPS for 10 h before qPCR analysis. (**E**) Effect of Rnf128 on chemotactic migration of macrophages. Representative images are shown (left) and the graphs show the relative migration rates in each group (right). Scale bars: 10 μm. (**F**) Sex-matched and age-matched WT (*n* = 7) and Rnf128-deficient mice (*n* = 7) intraperitoneally with LPS and the survival of mice was monitored every 2 h. (**G**) RNF128 deficiency promoted the activation of STAT5 after 12 h of LPS treatment. (**H**) Sex-matched and age-matched WT (*n* = 4), WT (*n* = 6) and Rnf128-deficient mice (*n* = 5) intraperitoneally with LPS or not for 12 h. Renal tissues were collected and subjected to qPCR analysis. The data are presented as the mean ± SEM. * *P* < 0.05; ** *P* < 0.01; *** *P* < 0.001

IL-3 is a hematopoietic growth factor closely associated with the intensity of immune responses. It has found that IL-3 influences the progression of inflammatory diseases such as inflammatory bowel disease (IBD) [2], sepsis [1], and viral infections [6]. IL-3 serves as a source of cytokine storm by promoting the survival, differentiation, proliferation, or recruitment of leukocytes [6, 25]. Considering the role of IL-3 in macrophage activation and RNF128 in degrading IL-3 receptor, we next investigate the functions of RNF128 in macrophage activation response to LPS. WT and *Rnf128*-deficient BMDMs were stimulated with LPS, and the expression levels of inflammatory and chemotactic factors were assessed by qPCR. The results indicated that *Rnf128* deficiency enhanced LPS-induced proinflammatory cytokines, such as *Il6*, *Tnfα*, *Ccl2* and *Kc* (Fig. 5A-D). BMDMs were subsequently exposed to LPS/IFNγ to mimic chronic inflammatory conditions. It was found that *Rnf128* deficiency promoted the expression of proinflammatory cytokines induced by LPS/IFNγ (Figure S3). Additionally, transwell migration assays were performed to assess the chemotaxis of macrophages. The deficiency of RNF128 promoted macrophage chemotaxis in response to LPS, and IL-3 further amplified the chemotaxis of macrophages under RNF128 deficiency (Fig. 5E). Additionally, we investigated the roles of Rnf128 in LPS-induced sepsis by injecting sex-matched and age-matched WT and Rnf128-deficient mice intraperitoneally with LPS. We observed that Rnf128-deficient mice were more susceptible to septic death than the wild-type mice following LPS stimulation (Fig. 5F). RNF128 deficiency promoted the activation of STAT5 after 12 h of LPS treatment (Fig. 5G). Consistently, *Rnf128* deficiency heightened the expression of LPS-induced proinflammatory cytokines, including *Il6*, *Tnfα*, *Ccl2* and *Il1-β*, in renal tissues (Fig. 5H). Collectively, these results indicated that *RNF128* knockout promotes the inflammatory process induced by LPS.

## Discussion

Ubiquitination is one of the primary mechanisms of post-translational modification in proteins, playing a crucial role in regulating protein degradation or activity. The primary mechanism for targeted protein degradation within the cell is accomplished by the ubiquitin-proteasome system (UPS) and autophagy-lysosomes [26]. Conventionally, ubiquitination modifications, particularly K48-linked ubiquitination, are commonly thought to lead to degradation by 26 S proteasome. Autophagic degradation is a meticulously regulated and selective cellular process in maintaining cellular homeostasis, adapting to stress and eliminating damaged cellular components [27]. The conventional viewpoint held that UPS and autophagy were two independently operating pathways for intracellular protein degradation. However, mounting evidence supports the notion that the two processes are closely interconnected. It has been found that the autophagy receptor p62 binds to polyubiquitinated substrates, anchoring them to the autophagosome for degradation [28]. E3 ubiquitin ligase HACE1 directly interacts with and catalyzes OPTN K48-linked polyubiquitination, ultimately resulting in the assembly of a large autophagic receptor protein complex by p62, facilitating the autophagic degradation pathway [29]. HECT ubiquitin E3 ligase NEDD4 binds to the active form of TBK1 to undergo K27-linked poly-ubiquitination, which is recognized by the cargo receptor NDP52 and result in the selective autophagic degradation [30]. Therefore, these studies collectively suggest a close association between ubiquitination modification and autophagy.

IL-3 signaling plays a critical role in hematopoiesis, immune cell activation, and cell survival, with its dysregulation or abnormal production associated with inflammatory diseases. Elevated plasma IL-3 levels in individuals with sepsis are correlated with increased mortality, even after adjusting for prognostic indicators [1]. Low plasma IL-3 levels in SARS-CoV-2-infected patients are associated with heightened disease severity, increased viral load, and elevated mortality, highlighting IL-3 as a potential early predictive marker for identifying at-risk individuals [6]. IL-3 levels are also increased in the cerebrospinal fluid (CSF) of multiple sclerosis (MS) patients, and its association with human relapsing-remitting MS (RRMS) exacerbates spinal cord inflammation, demyelination, and clinical severity in experimental autoimmune encephalomyelitis (EAE) [31]. IL-3Rα serves as the specific receptor for IL-3, making it a key target in the regulation of IL-3 signaling. The E3 ubiquitin ligase MARCH3 exerts a negative regulatory role in IL-3-triggered signaling by facilitating K48-linked polyubiquitination at position K377 of IL-3Rα and promoting its subsequent proteasomal degradation [32]. The hematopoietic GTPase RhoH acts as a negative regulator for IL-3-induced signals by controlling STAT activity and IL-3 Receptor α expression, ultimately resulting in the preferential activation of STAT1 and decreased cell proliferation in acute myeloid leukemia [33].

**Fig. 6:**
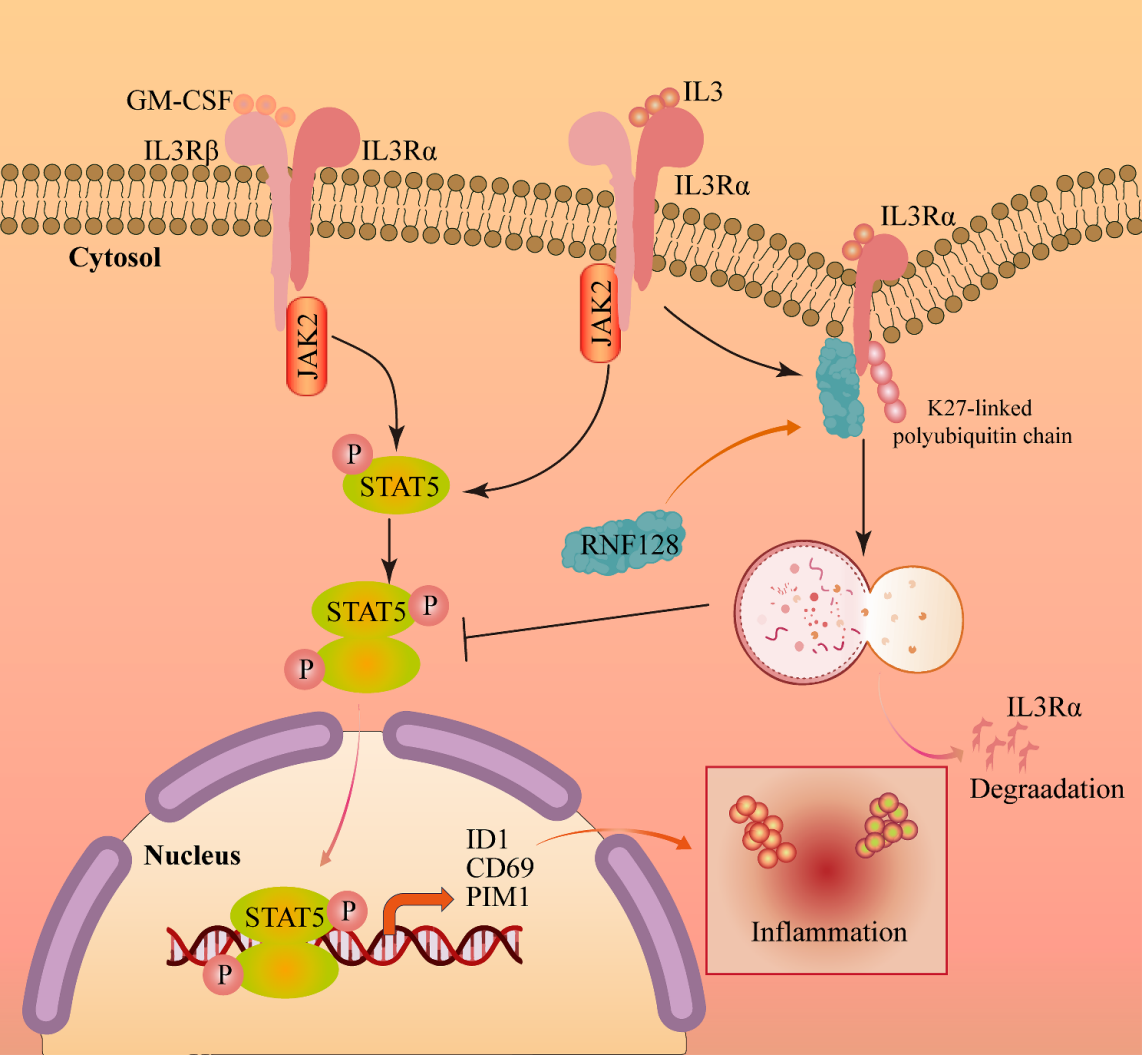
The schematic diagram illustrates the role of RNF128 in the IL3/STAT5 signaling pathway. The IL-3 signaling pathway relies on the IL-3 receptor complex, comprised of IL-3Rα, responsible for IL-3 binding specificity, and the common beta chain IL-3Rβ, shared with GM-CSF and IL-5 receptors. Upon IL-3 binding to its specific receptor, IL-3Rα initiates a cascade of downstream signaling events, involving the activation of JAK kinases, particularly JAK2. This activation subsequently leads to STAT5 phosphorylation, facilitating the transcription of genes like ID1, PIM1, and CD69. Notably, RNF128 demonstrates specific binding to IL-3 receptor alpha chain IL-3Rα, distinct from the common beta chain IL-3Rβ. The functional role of RNF128 is underscored as it promotes K27-linked polyubiquitination of IL-3Rα, culminating in its degradation through the lysosomal pathway. Consequently, RNF128 plays a crucial role in tempering excessive inflammatory responses

RING finger transmembrane-domain-containing protein 2 (RNFT2), also known as TMEM118, serves as a negative regulator of IL-3–dependent cellular responses by facilitating IL-3Rα ubiquitination and proteasomal degradation [34]. In our findings, we demonstrated that RNF128 functions as an inhibitory regulator of IL-3/STAT5 signaling, promoting the K27-linked polyubiquitination of IL-3Rα and subsequently facilitating its degradation via the lysosomal pathway (Fig. 6). We first identified the specific binding of RNF128 to IL-3Rα through the PA domain consequently inhibiting IL-3-induced STAT5 activation the chemotaxis of macrophages. This supports the notion that RNF128 suppresses inflammatory responses in acute infections independently of its classical role in T cells. Furthermore, RNF128 was found to alleviate LPS-induced acute lung injury by targeting TLR4 for degradation, which subsequently leads to the reduction of the NF-κB signaling pathway and pro-inflammatory cytokine production in the early stages of inflammation [15]. The TLR4 signaling rapidly initiates the inflammatory response by recognizing LPS, while IL-3 signaling cascade significantly amplifies the intensity of inflammation and the involvement of inflammatory cells to fuel a cytokine storm [1]. Consequently, RNF128 may represent a promising therapeutic target that exerts influence over both the initiation and the perpetuation phases of the inflammatory response. Additionally, we also firstly unveiled that IL-3Rα undergoes K27-linked polyubiquitination and subsequent degradation via the lysosomal pathway. These discoveries contribute to our understanding of the intricate regulatory mechanisms governing IL-3/STAT5 signaling, providing novel insights for the development of therapeutic interventions in inflammatory and infectious diseases.

## Electronic supplementary material

Below is the link to the electronic supplementary material.

Supplementary Material 1

Supplementary Material 2

Supplementary Material 3

## Data Availability

The main data supporting the findings of this study are available within the article or Supplementary Material, which further inquiries can also be directed to the corresponding authors.
