# Supplementary material for: E3 ubiquitin ligase RNF128 negatively regulates the IL-3/STAT5 signaling pathway by facilitating K27-linked polyubiquitination of IL-3Rα
Source: Cell Commun Signal. 2024 May 3;22:254. doi: 10.1186/s12964-024-01636-4 (PMC11067302; doi:10.1186/s12964-024-01636-4)
Supplement: Supplementary file 1 — Supplementary Material 1 [file 12964_2024_1636_MOESM1_ESM.docx]

Table S1 Peptides identified in the LC-MS/MS study

| **Protein IDs** | **Gene names** | **Fasta headers** | **Score** |
| --- | --- | --- | --- |
| Q14210 | LY6D | sp\|Q14210\|LY6D_HUMAN Lymphocyte antigen 6D OS=Homo sapiens OX=9606 GN=LY6D | 59.823 |
| P31947 | SFN | sp\|P31947\|1433S_HUMAN 14-3-3 protein sigma OS=Homo sapiens OX=9606 GN=SFN | 48.02 |
| P62195 | PSMC5 | sp\|P62195\|PRS8_HUMAN 26S proteasome regulatory subunit 8 OS=Homo sapiens OX=9606 GN=PSMC5 | 43.837 |
| P25788 | PSMA3 | sp\|P25788\|PSA3_HUMAN Proteasome subunit alpha type-3 OS=Homo sapiens OX=9606 GN=PSMA3 | 15.003 |
| P13010 | XRCC5 | sp\|P13010\|XRCC5_HUMAN X-ray repair cross-complementing protein 5 OS=Homo sapiens OX=9606 GN=XRCC5 | 13.848 |
| P62258 | YWHAE | sp\|P62258\|1433E_HUMAN 14-3-3 protein epsilon OS=Homo sapiens OX=9606 GN=YWHAE | 12.576 |
| A1A4S6 | ARHGAP10 | sp\|A1A4S6\|RHG10_HUMAN Rho GTPase-activating protein 10 OS=Homo sapiens OX=9606 GN=ARHGAP10 | 12.547 |
| Q9UL25 | RAB21 | sp\|Q9UL25\|RAB21_HUMAN Ras-related protein Rab-21 OS=Homo sapiens OX=9606 GN=RAB21 | 9.1629 |
| **P26951** | **IL3RA** | **sp\|P269511\|IL3RA_HUMAN IL-3 receptor subunit alpha OS=Homo sapiens OX=9606 GN=IL3RA** | **7.6156** |
| P28482 | MAPK1 | sp\|P28482\|MK01_HUMAN Mitogen-activated protein kinase 1 OS=Homo sapiens OX=9606 GN=MAPK1 | 7.1301 |
| Q9BU76 | MMTAG2 | sp\|Q9BU76\|MMTA2_HUMAN Multiple myeloma tumor-associated protein 2 OS=Homo sapiens OX=9606 GN=MMTAG2 | 7.0903 |
| Q52LW3 | ARHGAP29 | sp\|Q52LW3\|RHG29_HUMAN Rho GTPase-activating protein 29 OS=Homo sapiens OX=9606 GN=ARHGAP29 | 6.2776 |
| Q5TCX8 | MAP3K21 | sp\|Q5TCX8\|M3K21_HUMAN Mitogen-activated protein kinase kinase kinase 21 OS=Homo sapiens OX=9606 GN=MAP3K21 | 5.9989 |
| O00212 | RHOD | sp\|O00212\|RHOD_HUMAN Rho-related GTP-binding protein RhoD OS=Homo sapiens OX=9606 GN=RHOD | 5.7064 |
| O00206 | TLR4 | sp\|O00206\|TLR4_HUMAN Toll-like receptor 4 OS=Homo sapiens OX=9606 GN=TLR4 | 5.7031 |
| O95071 | UBR5 | sp\|O95071\|UBR5_HUMAN E3 ubiquitin-protein ligase UBR5 OS=Homo sapiens OX=9606 GN=UBR5 | 5.6942 |
