# Supplementary material for: E3 ubiquitin ligase RNF128 negatively regulates the IL-3/STAT5 signaling pathway by facilitating K27-linked polyubiquitination of IL-3Rα
Source: Cell Commun Signal. 2024 May 3;22:254. doi: 10.1186/s12964-024-01636-4 (PMC11067302; doi:10.1186/s12964-024-01636-4)
Supplement: Supplementary file 2 — Supplementary Material 2 [file 12964_2024_1636_MOESM2_ESM.docx]

**Figure S1**


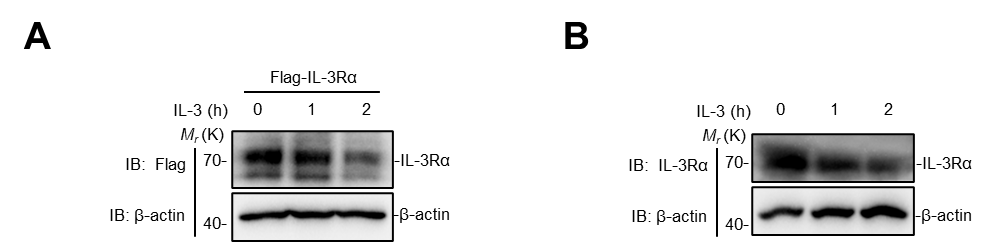


Figure S1 IL-3Rα undergoes degradation in a time-dependent manner following IL-3 treatment. (A) 293T cells were transfected with Flag-IL-3Rα for 10 hours and subjected to overnight starvation, followed by stimulation with IL-3 (20 ng/mL) for the indicated durations. (B) TF-1 cells underwent overnight starvation, followed by stimulation with IL-3 for the indicated durations. Subsequently, immunoblotting analysis was conducted for IL-3Rα and β-actin.

**Figure S2**


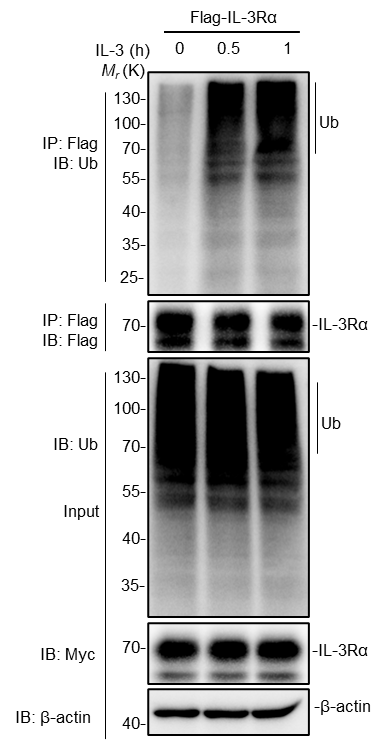


Figure S2 The level of ubiquitination for IL-3Rα significantly increases in response to IL-3 stimulation. 293T cells (~4 × 10^6^) were transfected with Flag-IL-3Rα for 10 hours and subjected to overnight starvation. Subsequently, the cells were stimulation with IL-3 for the indicated durations and subjected to ubiquitination analysis.

**Figure S3**

*
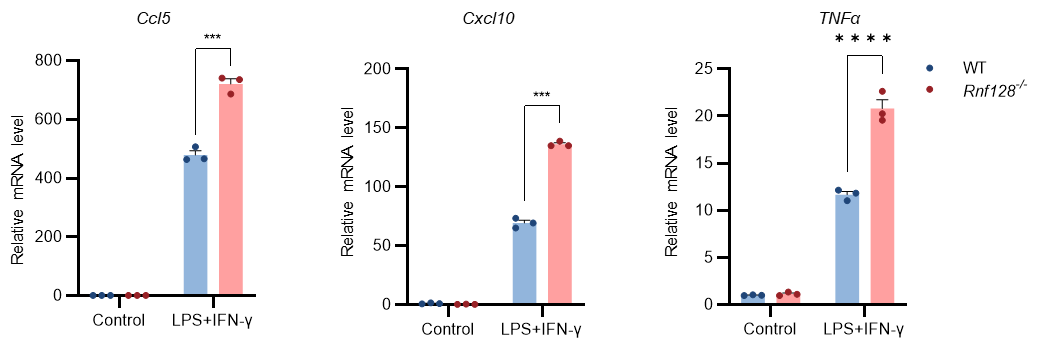
*

*Rnf128* deficiency promoted the expression of *Ccl5*, *Cxcl10* and *Tnfα* induced by LPS/IFNγ. Wild-type and *Rnf128*-deficient BMDMs were either untreated or treated with LPS/IFNγ (GM-CSF, 10 ng/ml; IFNγ, 25 U/ml) for 10 hours. The gene expression levels of *Ccl5*, *Cxcl10*, and *Tnfα* were measured by qPCR.
