# Supplementary material for: E3 ubiquitin ligase RNF128 negatively regulates the IL-3/STAT5 signaling pathway by facilitating K27-linked polyubiquitination of IL-3Rα
Source: Cell Commun Signal. 2024 May 3;22:254. doi: 10.1186/s12964-024-01636-4 (PMC11067302; doi:10.1186/s12964-024-01636-4)
Supplement: Supplementary file 3 — Supplementary Material 3 [file 12964_2024_1636_MOESM3_ESM.pdf]

Figure 1

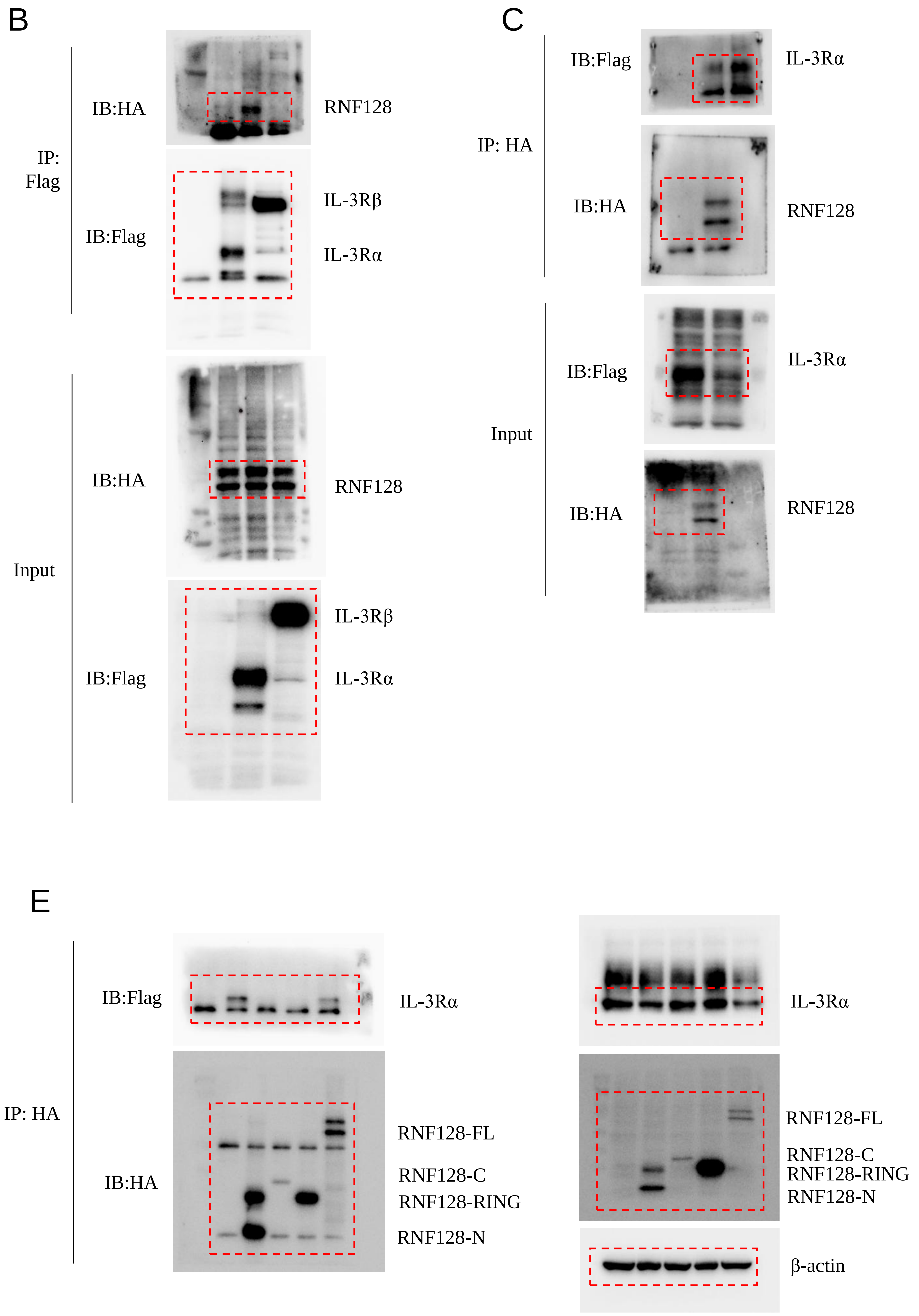

B
IP: Flag
IB:HA
RNF128
IB:Flag
IL-3Rβ
IL-3Rα
Input
IB:HA
RNF128
IB:Flag
IL-3Rβ
IL-3Rα
C
IP: HA
IB:Flag
IL-3Rα
IB:HA
RNF128
Input
IB:Flag
IL-3Rα
IB:HA
RNF128
E
IP: HA
IB:Flag
IL-3Rα
IB:HA
RNF128-FL
RNF128-C
RNF128-RING
RNF128-N
IL-3Rα
RNF128-FL
RNF128-C
RNF128-RING
RNF128-N
β-actin

Figure 2

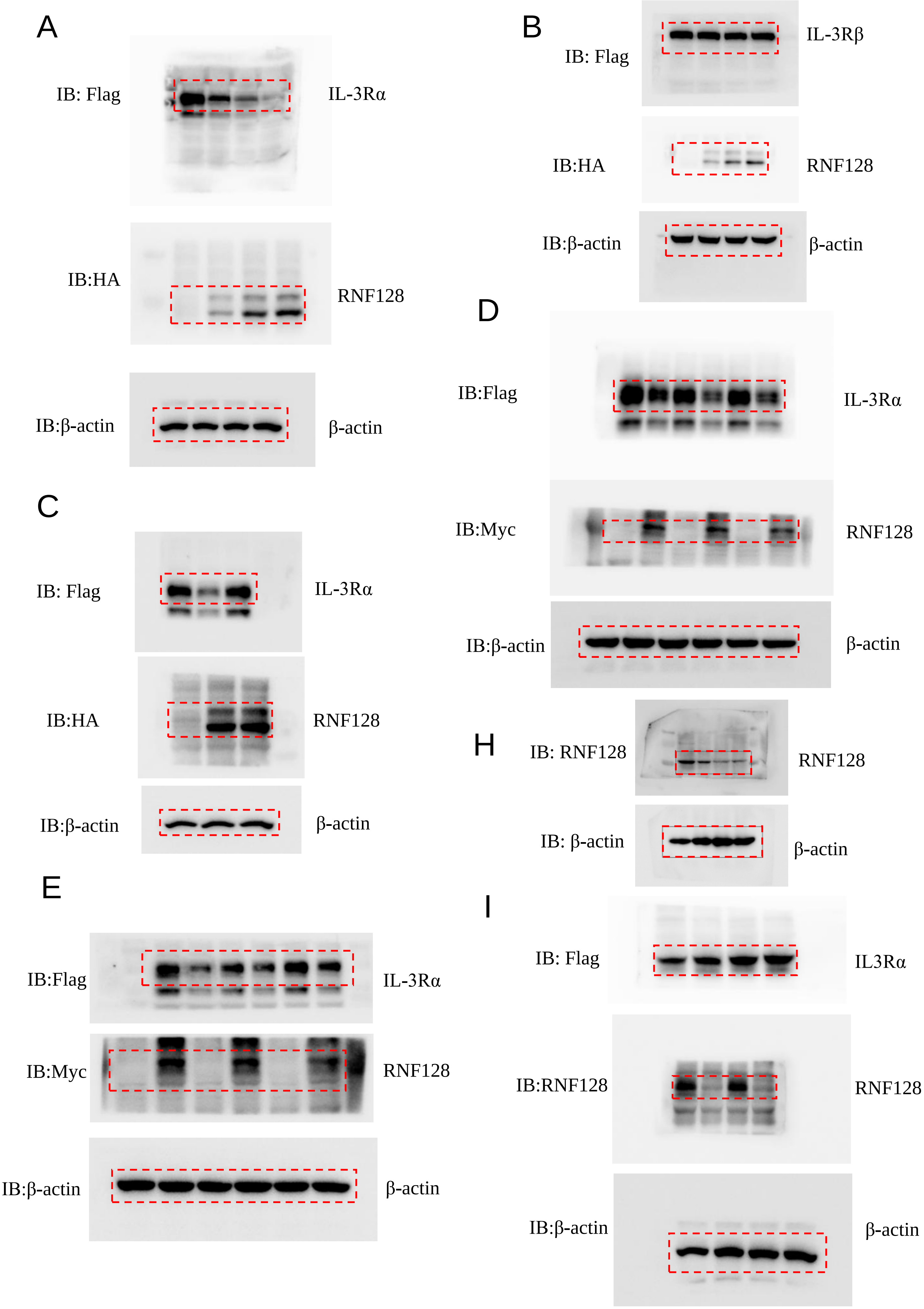
A
IB: Flag
IL-3Rα
IB:HA
RNF128
IB:β-actin
β-actin
B
IB: Flag
IL-3Rβ
IB:HA
RNF128
IB:β-actin
β-actin
C
IB: Flag
IL-3Rα
IB:HA
RNF128
IB:β-actin
β-actin
D
IB:Flag
IL-3Rα
IB:Myc
RNF128
IB:β-actin
β-actin
E
IB:Flag
IL-3Rα
IB:Myc
RNF128
IB:β-actin
β-actin
H
IB: RNF128
RNF128
IB: β-actin
β-actin
I
IB: Flag
IL3Rα
IB:RNF128
RNF128
IB:β-actin
β-actin

Figure 3

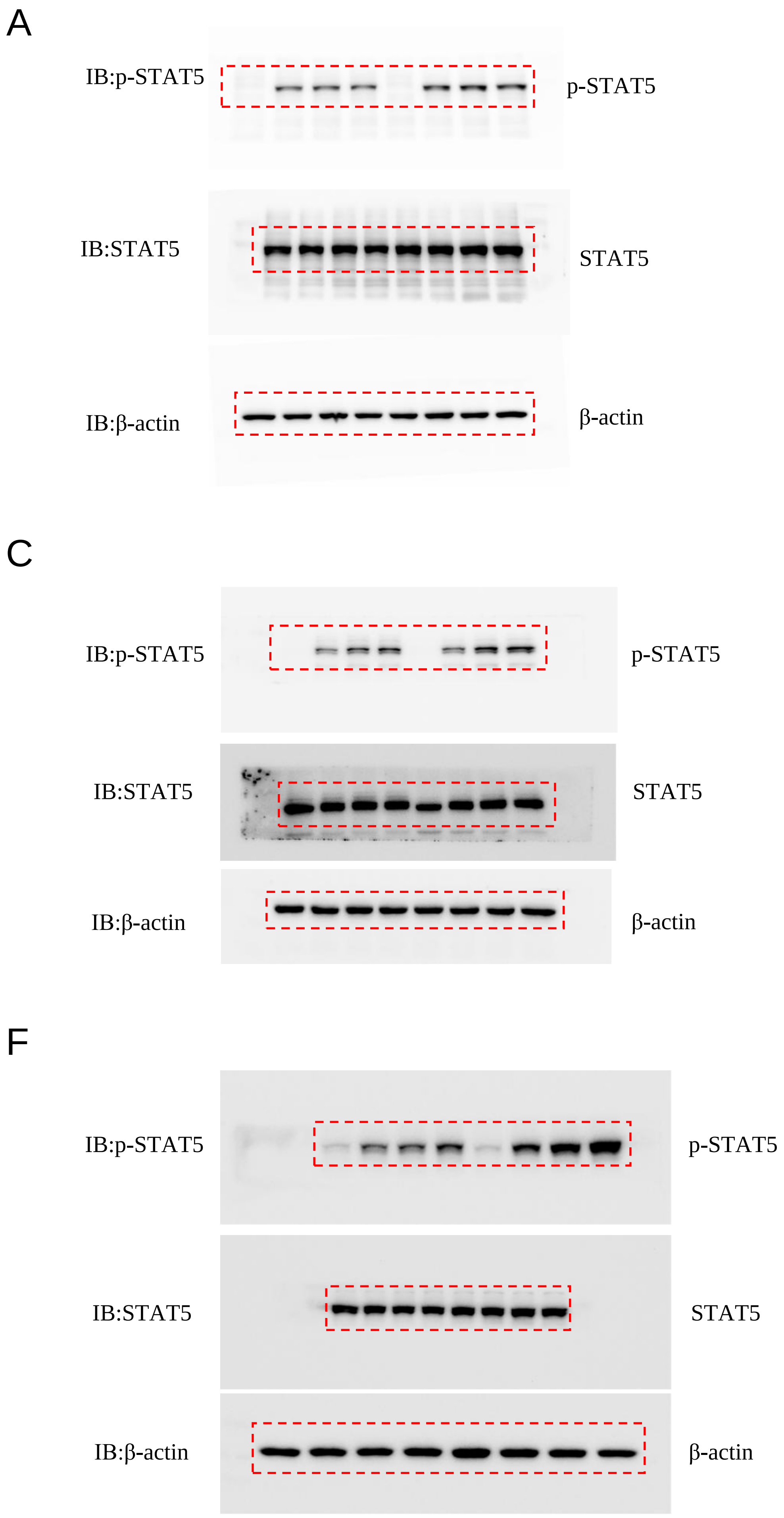
A
IB:p-STAT5
p-STAT5
IB:STAT5
STAT5
IB:β-actin
β-actin
C
IB:p-STAT5
p-STAT5
IB:STAT5
STAT5
IB:β-actin
β-actin
F
IB:p-STAT5
p-STAT5
IB:STAT5
STAT5
IB:β-actin
β-actin

Figure 4

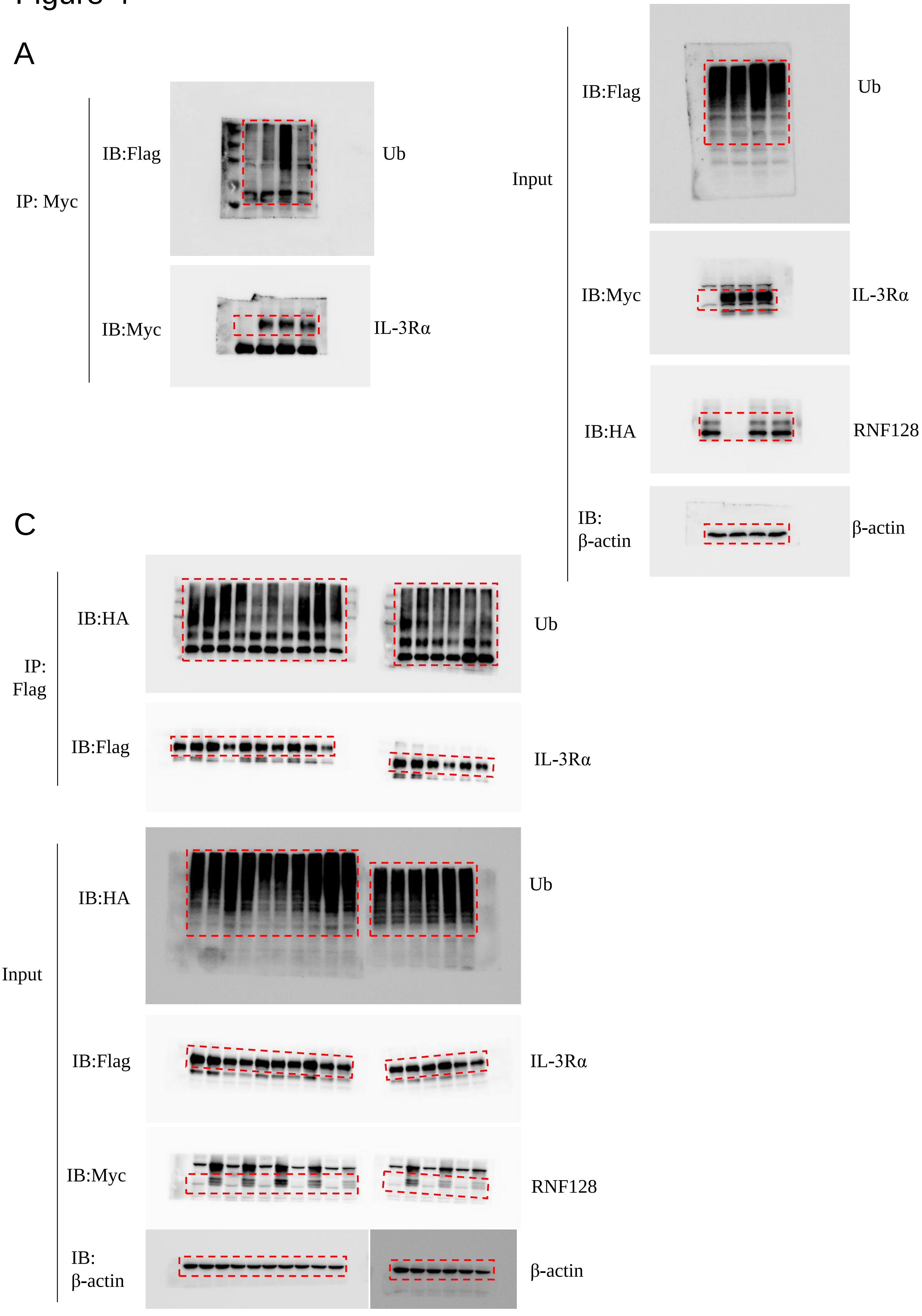

A
IP: Myc
IB:Flag
Ub
IB:Myc
IL-3Rα
Input
IB:Flag
Ub
IB:Myc
IL-3Rα
IB:HA
RNF128
IB:
β-actin
β-actin
C
IP:
Flag
IB:HA
Ub
IB:Flag
IL-3Rα
Input
IB:HA
Ub
IB:Flag
IL-3Rα
IB:Myc
RNF128
IB:
β-actin
β-actin

Figure 4

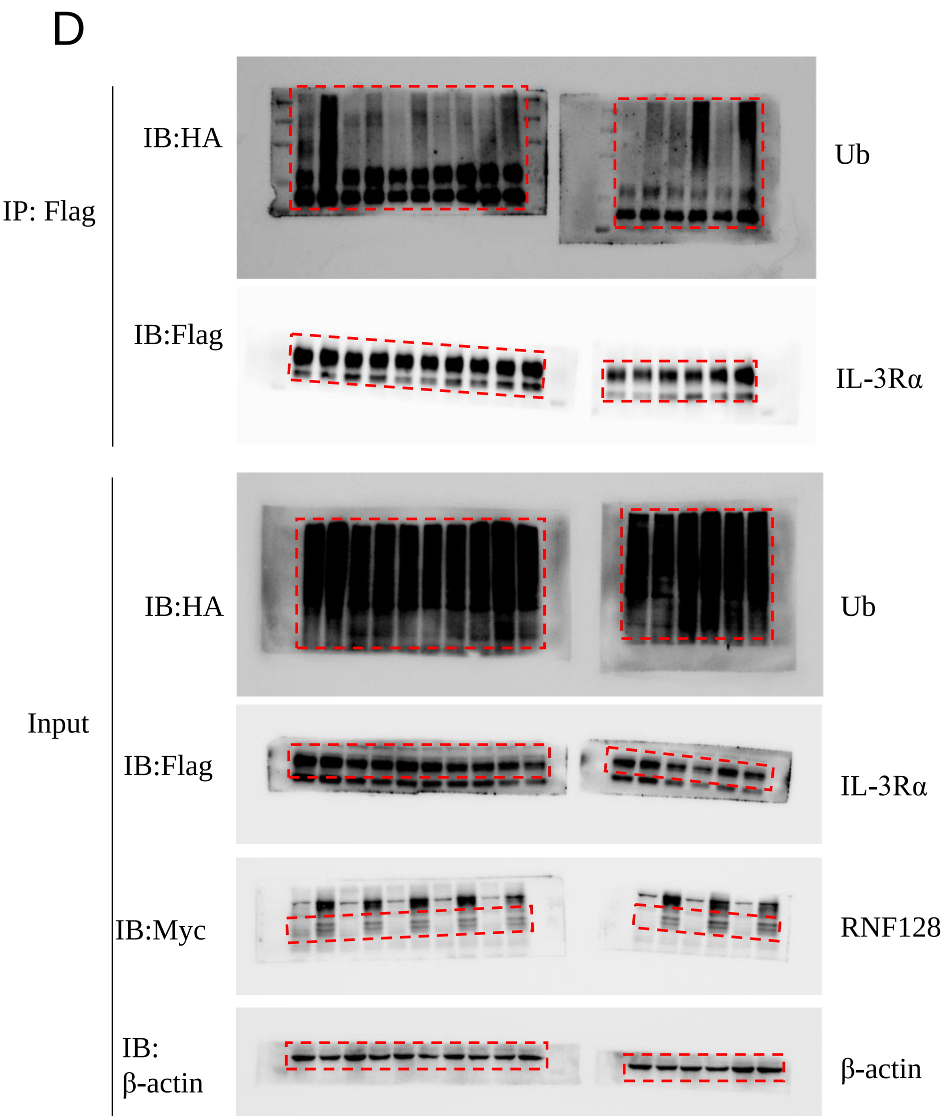

D
IP: Flag
IB:HA
Ub
IB:Flag
IL-3Rα
Input
IB:HA
Ub
IB:Flag
IL-3Rα
IB:Myc
RNF128
IB:
β-actin
β-actin

Figure 5

G

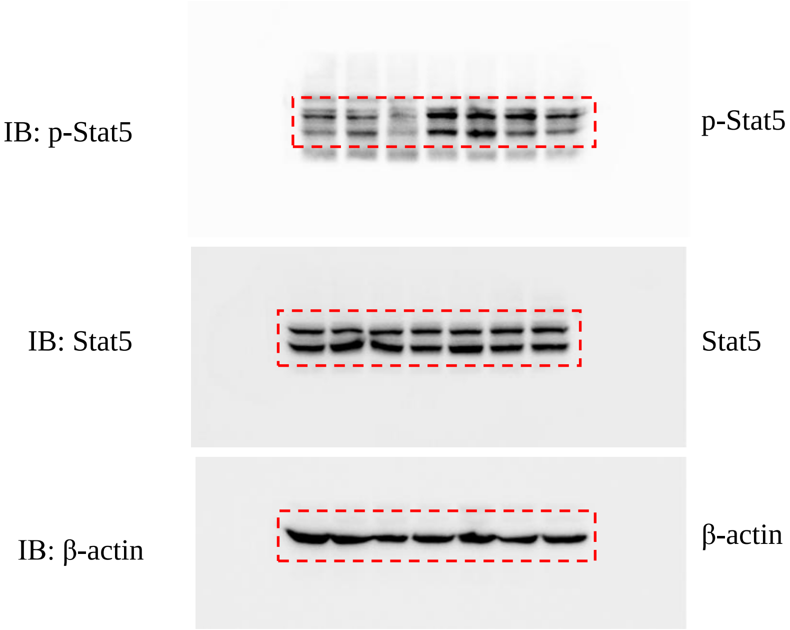

Figure S1

A

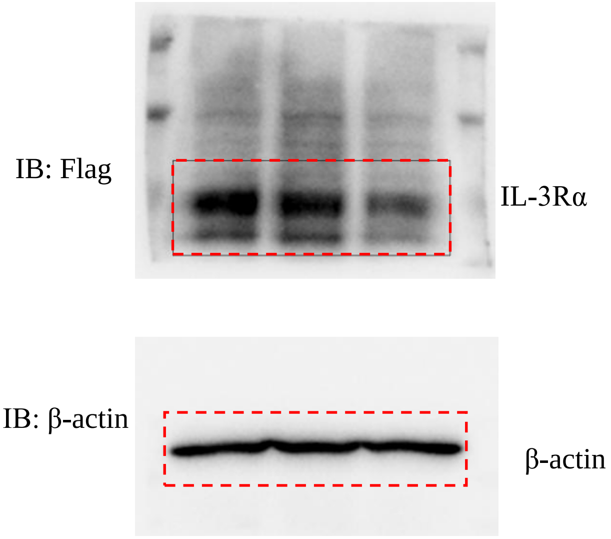

IB: Flag
IL-3Rα
IB: β-actin
β-actin

B

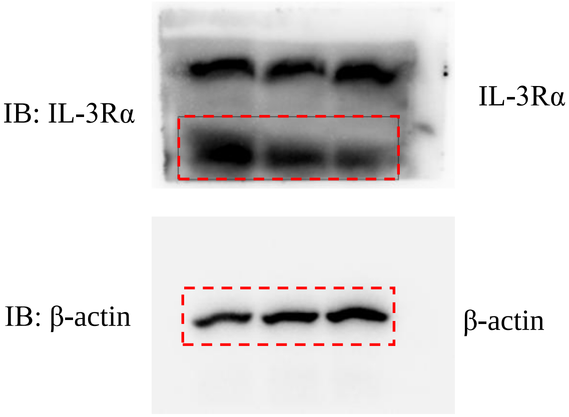

IB: IL-3Rα
IL-3Rα
IB: β-actin
β-actin

Figure S2

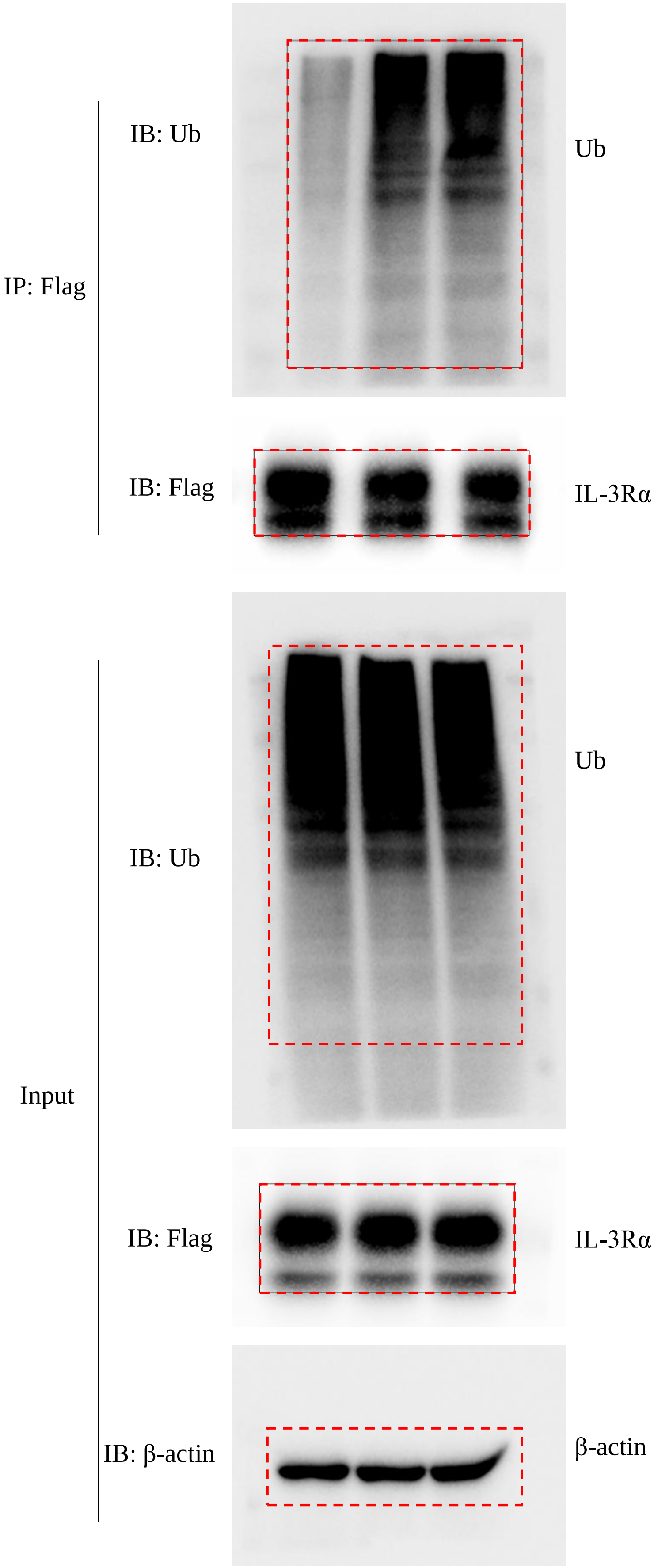
IP: Flag
IB: Ub
Ub
IB: Flag
IL-3Rα
Input
IB: Ub
Ub
IB: Flag
IL-3Rα
IB: β-actin
β-actin
